# Effectiveness and Safety of Electroconvulsive Therapy in Adolescents with Schizophrenia and Schizoaffective Disorder: A Clinical Case Series

**DOI:** 10.3390/jcm14248880

**Published:** 2025-12-15

**Authors:** Agnieszka Permoda-Pachuta, Piotr Obszański, Agata Makarewicz, Monika Dominiak, Adam Gędek

**Affiliations:** 1 Department of Neuroses, Personality Disorders and Eating Disorders, Institute of Psychiatry and Neurology, 02-957 Warsaw, Poland; 2 Department of Psychiatry, Psychotherapy and Early Intervention, Medical University of Lublin, 20-059 Lublin, Poland; 3 Institute of Psychiatry and Neurology, 02-957 Warsaw, Poland; 4 Third Department of Psychiatry, Institute of Psychiatry and Neurology, 02-957 Warsaw, Poland

**Keywords:** electroconvulsive therapy, adolescents, schizophrenia, schizoaffective disorder, treatment resistance, safety, effectiveness, case series

## Abstract

**Background:** Electroconvulsive therapy (ECT) is a well-established intervention for severe and treatment-resistant psychiatric disorders, yet its use in adolescents remains limited, particularly in Europe. Data on its safety and effectiveness in this population are scarce. This study aimed to evaluate the clinical outcomes and tolerability of ECT in adolescents diagnosed with schizophrenia and schizoaffective disorder. **Methods:** We conducted a retrospective observational case series of 22 adolescents (mean age 16.7 ± 1.3 years) treated with ECT between 2017 and 2024 at a university psychiatric department. Diagnoses included paranoid schizophrenia (n = 15), catatonic schizophrenia (n = 2), and schizoaffective disorder (n = 5). Symptom severity was assessed with the Positive and Negative Syndrome Scale (PANSS) before and after the ECT course. Adverse events were evaluated based on daily clinical monitoring and medical records. **Results:** The overall response rate, defined as ≥50% reduction in total PANSS score, was 82% (schizophrenia: 82%; schizoaffective disorder: 80%). Mean PANSS total score decreased from 158.0 ± 22.6 to 72.1 ± 20.7 (*p* < 0.0001). Improvements were most pronounced in the general psychopathology and positive symptom domains. No serious adverse events were observed. The most common transient side effects were headache (41%), memory complaints (27%), and somnolence (22%). **Conclusions:** ECT appears to be an effective and safe treatment option for adolescents with treatment-resistant schizophrenia and schizoaffective disorder. These findings add to the limited European evidence base and support considering ECT earlier in the treatment course of severe adolescent psychosis. Larger, prospective studies with long-term follow-up are warranted to confirm these results.

## 1. Introduction

Electroconvulsive therapy (ECT) remains one of the most effective biological treatments for severe psychiatric disorders. Since its introduction by Cerletti in 1938, ECT has been established as the gold-standard intervention for catatonia and treatment-resistant mood disorders, and it is also a valuable augmentation strategy in schizophrenia, particularly in clozapine resistance [1,2]. Advances in anesthesia and muscle relaxation have markedly improved its safety profile, yet utilization remains limited due to public stigma, negative media portrayals, and persistent misconceptions [3,4,5].

Clinical practice guidelines acknowledge the important role of ECT. The National Institute for Health and Care Excellence (NICE) and the Royal Australian and New Zealand College of Psychiatrists (RANZCP) recommend ECT for severe depression, mania, and catatonia, with RANZCP further including schizophrenia and schizoaffective disorder [6,7]. The American Psychiatric Association (APA) adopts an even broader stance, allowing for its use when clinically appropriate or when preferred by the patient [8]. The American Academy of Child and Adolescent Psychiatry (AACAP) specifies similar indications for minors—major depression, mania, schizophrenia, schizoaffective disorder, and catatonia—provided that symptoms are severe, persistent, and disabling, and that patients have failed to respond to at least two adequate pharmacological trials [9]. In contrast, European guidelines are typically more restrictive, often relegating ECT in children and adolescents to a “last resort” intervention [10].

Research and clinical practice confirm that despite the advances in psychopharmacology some patients fail to respond to medications and stand to benefit from ECT in both psychotic [11] and mood disorders [12]. Schizophrenia is a serious mental illness (SMI), which typically begins in the second and third decade of life. Early onset is often associated with greater functional impairment, which leads to the pressing need for effective treatments in the adolescent population [13]. Schizoaffective disorder (SAD) also belongs to the SMI group; however, detailed studies of the prevalence of diagnosis and its influence on functioning in the pediatric population are lacking. SAD includes the presence of both schizophrenic and affective symptoms, which do not warrant a diagnosis of schizophrenia or bipolar disorder. As in schizophrenia, electroconvulsive therapy has been successfully utilized in the treatment of patients with SAD [14]. Catatonia is a severe neuropsychiatric disorder affecting movement, speech, and complex behaviors, often involving autonomic and affective disturbances. In the International Classification of Diseases, Tenth Edition (ICD-10), it was classified as a subtype of schizophrenia, but in the more recent International Classification of Diseases, Eleventh Edition (ICD-11) and the Diagnostic and Statistical Manual of Mental Disorders, Fifth Edition, Text Revision (DSM-5-TR), it is an independent diagnosis. Electroconvulsive therapy is a first-line treatment in this disorder, with the additional benefit being the potential of ECT to ameliorate a disorder underlying the catatonia such as manic or depressive episode [15].

Electroconvulsive therapy was first performed on children and adolescents in 1941. Since then, it has remained in usage in the same indications as in the adult population [16]. However, due to the change in the conceptualization of psychiatric disorders over the course of the last 80 years, it is difficult to apply modern diagnostic labels to many patients described in the literature. The effectiveness of ECT is greatest in affective disorders and catatonia, followed by psychotic disorders. Walter and Rey (1997), after examining 46 adolescent patients treated with ECT, found that remission of symptoms occurred in 85% of patients with bipolar disorder (n = 11) and psychotic depression (n = 11), 100% with a manic episode (n = 3), and 28% with a schizophrenia spectrum disorder (SDD) (n = 5) [17]. Baeza et al. have reported in a series of thirteen cases of patients with SDD, aged 13–17, which mean PANSS decreased significantly at 6-month assessment compared to pre-ECT scores. Clinical response (20% or greater reduction in PANSS total scores) was achieved in 54% of patients [18]. A case series including 32 patients aged 12–17 from Germany in 2022 concluded that ECT was safe and effective regardless of the diagnosis [19]. A recent study on 14 patients under the age of 18 suffering from catatonia who did not respond to benzodiazepine treatment demonstrated the efficacy of ECT in this group of patients. Furthermore, the study revealed the efficacy of bilateral treatments, with six out of the twelve patients who received right unilateral treatments and one patient who underwent bifrontal treatments transitioning to bilateral treatments due to an absence of response, which resulted in an improvement [20].

Reports on the use of ECT in children and adolescents in Poland are extremely scarce. Smug et al. have published a case series consisting of five adolescents with treatment-resistant paranoid schizophrenia treated with bitemporal ECT in 2014. No serious adverse events were observed in any of the patients who achieved clinical improvement depending on the individual case. The duration of improvement, assessed as rehospitalization, varied individually [21]. A case report was presented of a 17-year-old female patient diagnosed with schizophrenia and seizures. The clinical outcome of bilateral ECT was satisfactory, with no occurrence of serious adverse events [22]. The Polish Psychiatric Association’s guidelines concerning the treatment of early-onset schizophrenia suggest that ECT is not frequently included in global recommendations, yet it is acknowledged by experts as a treatment modality that can be advantageous in specific cases. However, its application among children and adolescents remains underutilized [23].

This study provides new clinical data from a Central European population, representing the largest Polish case series of adolescents with schizophrenia spectrum disorders treated with ECT. As ECT in minors is rarely practiced and insufficiently documented in Europe, largely due to persisting concerns and hesitancy regarding its safety and potential effects on the developing brain, our findings offer unique regional real-world evidence and help to fill a notable gap in the existing literature on adolescent schizophrenia treatment. Against this background, the aim of this study was to evaluate the safety and effectiveness of ECT in adolescents with treatment-resistant schizophrenia or schizoaffective disorder.

## 2. Materials and Methods

### 2.1. Study Design and Settings

We conducted a retrospective, single-center, observational study at the I Department of Psychiatry, Medical University of Lublin. Patients were treated on the specialized Child and Adolescent Psychiatry Ward between 2017 and 2024.

### 2.2. Participants

All patients fulfilled ICD-10 diagnostic criteria for paranoid schizophrenia, catatonic schizophrenia, or schizoaffective disorder. Inclusion criteria to ECT were as follows: (1) agitation or aggression not responding to antipsychotic medication used within the recommended dosing range; (2) treatment resistance defined as lack of clinically significant improvement after at least two adequate antipsychotic trials of ≥8 weeks each; (3) catatonia with refusal of food intake. Exclusion criteria included acute medical illness, neurological disorders contraindicating ECT, or lack of informed consent.

The initial intention was to incorporate patients of neurodevelopmental age (up to 25 years of age) into the study; however, the analysis was conducted on patients up to the age of 19, which corresponds to the definition of adolescents (according to the WHO).

### 2.3. ECT Procedure

Before the initiation of electroconvulsive therapy, all patients underwent standard pre-treatment evaluations, including brain imaging (CT or MRI), EEG, and laboratory testing (complete blood count, electrolytes, liver and thyroid function tests, CRP, and prolactin). Each case was additionally consulted by a neurologist and a cardiologist to confirm the absence of contraindications. Patients and their legal guardians received oral and written information regarding the diagnosis, available treatment options, and the potential risks and benefits of ECT, after which written informed consent was obtained from legal guardians and from patients aged 16 years or older. In Poland, no authoritative guidelines on the practice of electroconvulsive therapy were in effect in the above-mentioned period.

ECT sessions were administered twice weekly, on Mondays and Fridays, using a bitemporal electrode placement with a brief-pulse constant-current device (Thymatron System IV, Somatics LLC, Venice, FL, USA). Anesthesia was induced with propofol (1–1.5 mg/kg), followed by muscle relaxation with succinylcholine (0.5 mg/kg) and oxygenation prior to stimulation. After each treatment session, patients were monitored by a psychiatrist through routine clinical interviews conducted before and after the procedure and during daily morning and evening assessments throughout the entire hospitalization period. At the end of the ECT course, the same set of laboratory tests performed at baseline was repeated.

### 2.4. Clinical Assessment

Patients were assessed using The Positive and Negative Syndrome Scale (PANSS), completed on the day before the first ECT session and 14 days after the last ECT session. PANSS is a medical scale developed originally to measure symptom severity in schizophrenia but has since then used in clinical trials of medications in other psychiatric diagnoses such as schizoaffective disorder [24]. Duration of untreated psychosis (DUP, in months) was obtained by clinical interview with the patient and parents or guardians [25]. Safety was assessed based on records of interviews performed by a specialist psychiatrist before and after each treatment session as well as every morning and evening for the whole length of the hospitalization period.

### 2.5. Statistical Analysis

STATISTICA PL Version 10.0 was used for statistical analysis. Differences between the baseline and endpoint PANSS scores encompassing total, positive symptoms, negative symptoms, and general psychopathology dimensions were assessed using Wilcoxon paired data test. Spearman’s rank correlation coefficient was calculated in order to assess the relationships between the total PANSS score and symptom domains. Treatment response was defined as a 50% reduction in the initial total PANSS score [26]. The level of statistical significance was set at *p* < 0.05.

## 3. Results

### 3.1. Patients Characteristics

Twenty-two patients (mean age 16.7 ± 1.3 years; male-to-female ratio 5:6) were included. Diagnoses were paranoid schizophrenia (n = 15), catatonic schizophrenia (n = 2), and schizoaffective disorder (n = 5). The mean duration of untreated psychosis was 9.9 ± 9.9 months. All patients had received at least two antipsychotic trials before ECT. Detailed characteristics are presented in Table 1.

Pathological changes in EEG pre-ECT were found in 81.4% of patients (N = 18). Abnormal brain scan findings pre-ECT were present in 18.2% of patients (N = 4). All EEG and MRI abnormalities were reviewed by physician prior to ECT initiation. None constituted a contraindication or required modification of the treatment. No significant laboratory abnormalities were observed prior to ECT. In laboratory tests performed post-ECT 18.2% of patients presented with elevated CRP levels. No other significant changes in laboratory parameters were observed post-ECT.

The number of ECT sessions per patient ranged from 10 to 12 (mean: 12.4). For each session, the electrical stimulus intensity was titrated individually, expressed as a percentage of the device’s maximum output (5–50%). Stimulation energy for individual seizures varied between 5 and 50% of the device output (mean: 26%). The mean seizure duration was 41 s. All patients received propofol (60–120 mg), succinylcholine (40–80 mg), and atropine (0.5–1 mg) for anesthesia induction, except for two sessions in one patient where etomidate (12–14 mg) was used instead of propofol. In ten patients, at least one ineffective seizure was recorded (defined as seizure duration < 20 s). Four patients experienced three such ineffective sessions, three patients experienced two, and one patient experienced a single ineffective seizure.

At the time of ECT, patients were receiving the following psychotropic medications: aripiprazole up to 30 mg/d (n = 11), risperidone up to 4 mg/d (n = 10), clozapine up to 400 mg/d (n = 7), olanzapine up to 20 mg/d (n = 4), haloperidol up to 3.5 mg/d (n = 3), sertraline up to 50 mg/d (n = 3), lurasidone up to 74 mg/d (n = 2), quetiapine up to 300 mg/d (n = 2), venlafaxine up to 150 mg/d (n = 1), zuclopenthixol up to 50 mg/d (n = 1), and lithium carbonicum up to 500 mg/d (n = 1).

### 3.2. Effectiveness—PANSS Scores

**Figure 1:**
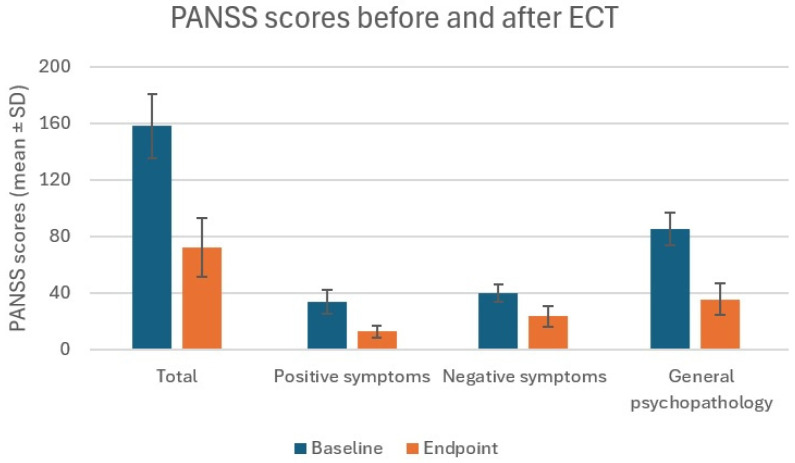
PANSS scores (mean ± SD).

Overall response rate was 82% (N = 18). In the schizophrenia group, the response rate was 82% (N = 14) and in the schizoaffective disorder group 80% (N = 4). PANSS scores are presented in Table 2 and Figure 1.

Total PANSS baseline score mean was 158 ± 22.6 (points) and total endpoint was 72.1 ± 20.7. Positive symptoms score at baseline was 33.8 ± 8.5 and 12.8 ± 4.0 after ECT. Negative symptoms score was 40.0 ± 6.1 pre-ECT and 23.7 ± 7.5 post-ECT. General psychopathology score was 85.2 ± 11.6 at baseline and 35.7 ± 11.0 at endpoint. The Wilcoxon test values for every pair of scores were 4.11 with *p* < 0.0001. No significant differences were found between any endpoint scored between schizophrenia and schizoaffective disorder groups (*p* > 0.05). No association has been found between the PANSS scores and laboratory tests, brain abnormalities found in imaging, EEG abnormalities, and DUP. The patient group was small, and the distribution of diagnoses was uneven, factors that will be underscored in the paragraph on study limitations.

### 3.3. Safety and Adverse Events

Adverse events reported by patients and recorded in the documentation were headache (N = 9, 41%), subjective memory problems (N = 6, 27%), somnolence (N = 5, 22%), nausea and vomiting (N = 2, 9%), and concentration impairment (9%). No cases of postictal confusion lasting beyond the recovery room period, cardiovascular instability, prolonged seizures, or musculoskeletal pain were recorded. All side effects resolved before the end of the hospitalization period and did not require any additional interventions. No serious adverse events were observed. All patients completed the full course of treatment.

## 4. Discussion

In this study we examined the effectiveness and safety of electroconvulsive therapy in adolescent population at an academic medical center. We found that ECT led to improvement in all symptom domains measured by PANSS, with the greatest change in general psychopathology and the smallest in negative symptoms. General psychopathology category included the following symptoms: somatic concern, anxiety, guilt feelings, tension, mannerisms and posturing, depression, motor retardation, uncooperativeness, unusual thought content, disorientation, poor attention, lack of judgment and insight, disturbance of volition, poor impulse control, preoccupation, and active social avoidance. Negative symptoms consist of blunted affect, emotional withdrawal, poor rapport, passive–apathetic social withdrawal, difficulty in abstract thinking, lack of spontaneity and flow of conversation, and stereotyped thinking. Factors most closely associated with the total PANSS score changes were general psychopathology and positive symptom scores. This possibly indicates that ECT exerts greater influence on the symptoms grouped in these domains than on the negative symptoms [27]. This pattern reflects the broad-spectrum neurobiological effects of ECT, which modulate large-scale cortical–subcortical network functioning and global arousal states, rather than selectively targeting dopaminergic mechanisms associated with positive psychotic symptoms. Symptoms such as anxiety, behavioral dysregulation, emotional tension, and attention or impulse control disturbances are particularly sensitive to this mechanism and may therefore improve more substantially. Positive symptoms also showed marked reduction in our cohort, but the relatively greater improvement in the general psychopathology domain likely reflects the multidimensional regulatory effects of ECT on brain network functioning rather than a narrow antipsychotic effect.

Patients treated with ECT at I Department of Psychiatry, Lublin Medical University, were diagnosed with either schizophrenia or schizoaffective disorder in line with the most common indications for the procedure found in large-scale studies in Sweden [28], the United States [29], and China [30]. However, in all of these studies, mood disorders outnumbered psychotic disorders, as indicated in Sweden (77% vs. 11%) and the US (65.6 % vs. 23.1%), with a smaller difference observed in the Chinese population (47.4% vs. 29.6%). The lack of mood disorder patients treated with ECT at our medical center can be attributed to the small number of cases treated in the years covered by this case series. It is noteworthy to add that better overall effects are achieved when ETC is utilized in patients with affective disorders, and this study was mainly concerned with treatment-resistant patients with a diagnosis from the schizophrenia group.

Response rates were similar to the ones reported in a recent Turkish retrospective study—93% in schizophrenia based on PANSS (responders were defined as having a 50% reduction of the initial PANSS score), 72% in bipolar disorder and depressive disorder based on the Hamilton Depression Rating Scale, and 90% in bipolar disorder based on the Young Mania Rating Scale [31]. In a Chinese study on the use of ECT in adolescents, the response rates using Global Clinical Impressions—Improvement scale showed the response rates in schizophrenia were 65.61%, 69.95% in bipolar disorder, and 78.57% in major depressive disorder [32]. In a different study of ECT in adolescents with schizophrenia from China, where the response rate was defined as a 20% reduction of PANSS scores in comparison to the initial evaluation, similar results were found—79.3% [33]. From the above-mentioned studies, it is possible to infer that ECT exerts a positive influence more often in bipolar disorder and schizophrenia than in schizoaffective disorder. In subsequent studies, it may be beneficial to evaluate the reduction in individual symptom clusters, independent of diagnosis.

Adverse events experienced by patients in our study were consistent with those reported in other centers, albeit with different rates of occurrence—with nausea (15.7%) and headache (13.7%) being most common in one study [34]. All side effects observed in our study were transient and completely subsided before discharge. The fact the side effects are mild and self-limiting is in line with a recent systematic review on the subject of adverse events in electroconvulsive therapy [35]. One of the most common adverse events following ECT reported in the literature are memory problems. In our study, side effects were evaluated on the basis of complaints reported in a clinical interview with a psychiatrist and no clinical scales were used. In a Swedish study on subjective memory complaints after ECT, 26% of patients reported such an adverse event, which is in line with our findings were only a minority of patients reported memory problems (27%) [36]. Studies of children and adolescents treated with ECT including a long-term follow-up have reported that cognitive side effects seem to be short-lasting and self-limiting. A study of 10 adolescents treated with ECT for severe mood disorder assessed cognitive function using Mini-Mental State, attention section of the Wechsler Memory Scale—Revised, and the California Verbal Learning Test after an average of 3.5 years. No difference was found between the study group and 10 healthy controls who completed the same tests [37]. Using a large battery of cognitive function tests, Ghaziuddin et al. assessed 7 adolescent patients before, a few days after concluding ECT and after 8 months. No deterioration of concentration, attention, verbal and visual memory, or verbal fluency was found at 8 months after the procedure [38]. In a study comparing efficacy and safety of ECT with antidepressants in adolescents with major depressive disorder and strong suicidal ideation, the Repeatable Battery for the Assessment of Neuropsychological Status (RBANS) was utilized in the measurement of cognitive function pre-ECT and at 2 and 6 weeks post-ECT. No difference was found between the two groups at 6 weeks in terms of cognitive function [39].

To our knowledge, this is the largest report of adolescent patients treated with ECT in Poland to date. This study adds to the growing body of evidence supporting the efficacy and safety of ECT in adolescents with severe, treatment-resistant psychiatric disorders. Despite robust clinical effectiveness, ECT remains underutilized and is often relegated to a last-line option in guidelines and clinical practice [10,24]. Our findings underscore the need for further systematic research, particularly within European populations, where data are scarce and most available evidence originates from the United States and Australia [10].

### Limitations

Important limitations to our study include the following: the small number of patients with a diagnosis of schizoaffective disorder, lack of long-term follow-up in terms of both functional improvement and potential late adverse effects, lack of comprehensive assessment of cognitive functions, and the increasing of medication doses simultaneously with ECT treatment. The study was seriously affected by the COVID pandemic from 2020 onwards and the interruptions it caused to standard care with the introduction of social distancing.

An important limitation of the present study is the lack of systematic long-term follow-up allowing for assessment of the durability of clinical improvement after ECT. Due to the retrospective design of the study and disruptions in continuity of care associated with the COVID-19 pandemic, information regarding time to relapse, subsequent treatment trajectories, and long-term functional outcomes could not be reliably obtained from the medical records. Given that the question of sustained efficacy and relapse prevention after ECT in adolescent treatment-resistant schizophrenia remains largely unanswered in the existing literature, future prospective studies should systematically monitor post-discharge functioning, including return to school, social reintegration, maintenance of family living, symptom recurrence, and time to rehospitalization. Such data will be essential for establishing whether ECT offers not only an acute therapeutic effect but also contributes to long-term stabilization and improved functional prognosis.

## 5. Conclusions

Electroconvulsive therapy appears to be an effective and safe method of treatment for adolescent patients with both psychotic and mood disorders. Large-scale prospective trials are necessary to obtain greater level of confidence in this regard, although their design and execution remain problematic. ECT should be considered as a viable treatment option early on in patients with high-severity symptoms and no satisfactory response to pharmacological treatment.

## Figures and Tables

**Table 1:** Patients characteristics (n = 22).

Patients Characteristics	
16.7 ± 1.3 (years)	Mean age
5:6	Male-to-female ratio
9.9 ± 9.9 (months)	Mean duration of untreated psychosis
15	Diagnosis of paranoid schizophrenia
2	Diagnosis of catatonic schizophrenia
5	Diagnosis of schizoaffective disorder
Every patient was treated with medications for at least 14 days before the implementation of ECT	Medications
All patients received dopamine, serotonin, and norepinephrine antagonists	
During the course of ECT treatment, 6 patients had a simultaneous medication dose increase ^1^	

^1^ Three patients with a diagnosis of paranoid schizophrenia (patient I—aripiprazole, II—risperidone, III—haloperidol, lurasidone), two patients with a diagnosis of catatonic schizophrenia (I—aripiprazole, II—clozapine), and one patient with a diagnosis of a schizoaffective disorder (venlafaxine).

**Table 2:** PANSS scores (mean ± SD).

PANSS Scores	Baseline	Endpoint	*p*-Value
Total	158 ± 22.6	72.1 ± 20.7	*p* < 0.0001
Positive symptoms	33.8 ± 8.5	12.8 ± 4.0	*p* < 0.0001
Negative symptoms	40.0 ± 6.1	23.7 ± 7.5	*p* < 0.0001
General psychopathology	85.2 ± 11.6	35.7 ± 11.0	*p* < 0.0001

## Data Availability

The data presented in this study are available upon request from the corresponding author. The data are not publicly available due to privacy or ethical concerns.
